# Dosage of Pimobendan in Domestic Cats

**DOI:** 10.1155/vmi/8218462

**Published:** 2026-10-14

**Authors:** Edinete Lúcio Pereira, Luana Thamilis Alves Bezerra, Thiago Da Silva Brandão, Rosangela Maria Nunes Da Silva, Almir Pereira De Souza

**Affiliations:** ^1^ Graduate Program in Animal Science and Health, Federal University of Campina Grande (UFCG), Av. Universitária S/n-Santa Cecília, Patos 58.708-110, Paraiba, Brazil, ufcg.edu.br; ^2^ Academic Unit of Veterinary Medicine, UFCG, Av. Universitária S/n-Santa Cecília, Patos 58.708-110, Paraiba, Brazil, ufcg.edu.br

**Keywords:** cardiopathic, dosage, felines, healthy, inodilator

## Abstract

**Background:**

Available evidence suggests that pimobendan may provide clinical benefits in the treatment of heart diseases in cats. However, its dosage is based on studies conducted in dogs, raising concerns due to differences in plasma concentrations between species.

**Review Question:**

What is the dosage of pimobendan used in domestic cats, either healthy or with heart disease?

**Methods:**

Searches in the PubMed, Web of Science, Scopus, and ScienceDirect databases were conducted between October and November 2024, a period corresponding to the execution of the search strategy. Full‐text articles were included without restrictions regarding language, country, or year of publication, while gray literature was excluded.

**Results:**

Out of 371 articles screened, 15 were excluded due to duplication or lack of relevance, resulting in 10 studies being included in the final review. Pimobendan dosages ranged from 0.125 mg/kg to 0.5 mg/kg, with oral administration being predominant, typically every 12 h. Some studies used single doses to evaluate the drug’s pharmacodynamics. Beneficial effects observed included improved cardiac output, enhanced left ventricular function, and reduced risk of thrombi. Common adverse effects were anorexia, vomiting, lethargy, hypotension, and arrhythmias, particularly tachycardia (4/10). The studies showed some risk of bias, such as lack of detailed information on sex, weight, and adverse effects in cats, heterogeneity in study populations, variability in pimobendan doses, and insufficient control of cardiac disease conditions.

**Conclusion and Clinical Relevance:**

Pimobendan was well tolerated in cats, with only isolated and no clinically relevant adverse effects reported, at doses ranging from 0.24 to 0.5 mg/kg or 1.25 mg/cat, with oral administration every 12 h being the recommended regimen.

## 1. Introduction

Pimobendan is a compound derived from benzimidazole‐pyridazinone, classified as an inodilator due to its inotropic and vasodilatory properties. The drug is metabolized in the liver, where it is converted into an active metabolite called O‐desmethylpimobendan [1]. In dogs, the drug’s bioavailability when administered orally is approximately 70%, whereas in cats, the elimination half‐life is nearly three times longer, and the plasma concentration can be up to ten times higher (34.5 ± 6.59 ng/mL vs. 3.09 ± 0.76 ng/mL), suggesting significant pharmacokinetic differences between species [2]. In healthy cats, pimobendan is rapidly absorbed, with the onset of action observed approximately 18 min post‐administration [3].

Regarding contraindications, caution is recommended when administering pimobendan to cats with severe dynamic left ventricular outflow tract obstruction (DOLVOT). Although no evidence of cardiotoxicity or significant extracardiac side effects has been found, some adverse effects, such as salivation, vomiting, diarrhea, anorexia, and constipation, have been observed in other studies [1, 3]. Furthermore, a dose of 1.25 mg/cat administered every 12 h orally did not cause significant changes in heart rate, blood pressure, or glomerular filtration rate, suggesting that pimobendan does not exert adverse hemodynamic effects in healthy cats [4].

Pimobendan has demonstrated potential therapeutic benefits in cats with cardiac disease. Although a retrospective study reported prolonged survival of approximately 626 days in treated cats compared with untreated cats [5], subsequent prospective clinical studies have not consistently confirmed this finding. The recommended dosage has been based on studies conducted in dogs, raising questions about its suitability for cats, considering the differences in plasma concentrations between the species. Moreover, the dose is not weight‐adjusted despite individual variability [3]. Therefore, determining the optimal dosage for cats remains a challenge.

The treatment of heart failure in cats is complex and continues to be a significant challenge for veterinarians. For this reason, the search for new treatments and combinations of therapies that can improve the quality of life and survival of feline patients is crucial. Considering the physiological differences between dogs and cats, additional studies are necessary to establish the correct dosage of pimobendan in cats and provide more accurate therapeutic guidance [6]. In this context, the objective of this systematic review was to identify and synthesize the pimobendan dosages reported for domestic cats with or without cardiovascular disease, as well as the associated clinical outcomes, cardiovascular findings, and adverse effects. Additionally, this review sought to identify knowledge gaps that may inform future studies aimed at establishing evidence‐based dosing recommendations for feline patients.

## 2. Materials and Methods

### 2.1. Study Design

This study is a systematic literature review following the methodological recommendations outlined by the Preferred Reporting Items for Systematic Reviews and Meta‐Analyses (PRISMA). The PICO strategy (Patient, Intervention, Comparison, and Outcomes) was employed, as follows:

• Review question: What evidence is available regarding the use and dosages of pimobendan in domestic cats with or without cardiovascular disease?
• Population: Domestic cats (*Felis catus*) with no restrictions on sex, age, or breed.
• Intervention: Use of pimobendan, regardless of dose, route of administration, or treatment duration, in the following scenarios:
  ◦ Experimental treatment in healthy cats
  ◦ Experimental treatment in cardiopathic cats
  ◦ Non‐experimental clinical treatment in healthy cats
  ◦ Non‐experimental clinical treatment in cardiopathic cats
• Intervention details:
  ◦ Therapeutic doses used
  ◦ Frequency of administration
  ◦ Routes of administration
  ◦ Observed cardiovascular effects
  ◦ Adverse effects
• Comparison: Dosages yielding the best cardiovascular effects without adverse effects in healthy and/or cardiopathic cats, compared to dosages with no improvement in cardiac function or causing adverse effects.
• Outcomes: All therapeutic results obtained from the dosages of pimobendan used in healthy and/or cardiopathic cats.

### 2.2. Search Strategies, Eligibility, and Exclusion Criteria

Based on predetermined inclusion criteria, the search was conducted between October and November 2024, with the last search performed on November 11, 2024. Searches were carried out in the National Library of Medicine (PubMed), Web of Science, Scopus, and ScienceDirect databases using the keywords “cat OR feline” AND “pimobendan” AND “dose OR dosage,” tailored to each platform’s search capabilities. Identified documents with titles and abstracts were saved, downloaded in “BibTeX” format, and exported to a reference management software (Mendeley) for further selection.

Inclusion criteria

• Results obtained from the treatment of healthy cats with pimobendan
• Results obtained from the treatment of cardiopathic cats with pimobendan
• Frequency of administration
• Route of administration
• Doses used
• Patient survival
• Full scientific articles and case reports describing prescribed dosages
• Studies published in English, Spanish, and/or Portuguese
• Open access articles
• No restrictions on country of origin or publication year

Exclusion criteria

• Association with other positive inotropic drugs
• Studies in other species (e.g., humans, canines, wild cats, and rodents)
• Studies that did not report pimobendan dosage or did not provide data relevant to the objectives of this review, including clinical outcomes, cardiovascular findings, frequency or route of administration, or adverse effects
• Studies without original data
• Book chapters and encyclopedias
• Publications with restricted access
• Conference proceedings.

### 2.3. Study Selection and Data Extraction

Duplicates in the reference manager (Mendeley) were removed, retaining only unique articles to avoid repetition. Subsequently, two researchers (Edinete Lúcio Pereira and Almir Pereira De Souza) independently selected studies based on titles and abstracts. Articles not meeting eligibility criteria were excluded after full‐text review, resolving any disagreements through consensus. Extracted data, such as authors, publication year, titles, and sources, were organized in a Microsoft Excel spreadsheet.

Cardiovascular findings, clinical outcomes, and adverse events were extracted exactly as reported in the included primary studies and were synthesized descriptively, preserving the terminology, outcome definitions, and assessment methods adopted by the original authors. Owing to the heterogeneity of study designs and methodological approaches, no attempt was made to standardize or reinterpret the reported findings, and the original classifications were maintained whenever explicitly reported.

### 2.4. Methodological Quality Assessment

A narrative review was conducted, encompassing all selected studies. The quality, quantity, and consistency of evidence across studies were independently assessed by two authors. Methodological risk of bias in the selected studies was evaluated independently by the same authors (Edinete Lúcio Pereira and Almir Pereira De Souza), reaching consensus on risk ratings categorized as uncertain, high, or low. Only studies with a low risk of bias were considered reliable, and none were excluded. The methodological protocol was developed by the authors prior to the study and was not submitted to registration in prospective databases.

### 2.5. Data Analysis

Qualitative results were analyzed using descriptive statistical methods, presenting absolute and percentage distributions of the data.

## 3. Results

The search strategy across PubMed, Web of Science, Scopus, and ScienceDirect identified 371 records. Whenever available, database‐specific filters consistent with the predefined eligibility criteria (e.g., species, publication type, language, and open‐access availability) were applied within each platform before exporting the search results. As a result of this initial refinement, 156 ineligible records were excluded, including studies conducted in other species, book chapters, and publications with restricted access. The remaining records were then exported to Mendeley, where duplicate articles (*n* = 15) were removed, leaving 200 studies for title screening.

After title and abstract screening according to the predefined eligibility criteria, 11 studies were selected for full‐text assessment. One study was subsequently excluded because it evaluated only platelet‐related variables and did not provide data relevant to the objectives of this review. Consequently, 10 studies met all eligibility criteria and were included in the systematic review (Figure 1).

**FIGURE 1:**
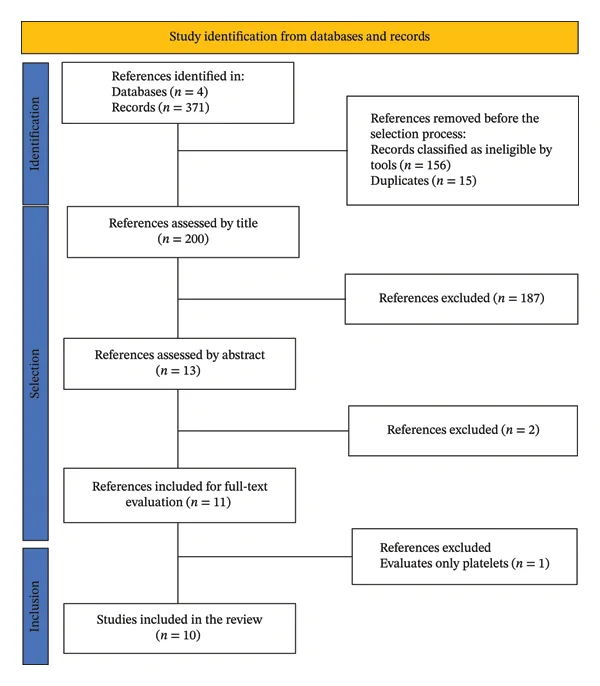
Flowchart of identification and selection of articles for the systematic review on the dosage of pimobendan in healthy and/or cardiopathic domestic cats.

### 3.1. Characteristics of the Included Studies

The main epidemiological characteristics of the studies are presented in Table 1. Regarding geographical distribution, the studies selected according to the inclusion criteria for the qualitative synthesis on pimobendan dosage in domestic cats are primarily from the United States (8/10), with a smaller proportion from Japan (2/10). The study designs were case–control (6/10), retrospective cohort (3/10), and prospective (1/10).

**TABLE 1:** Main epidemiological characteristics of studies on pimobendan dosage in healthy and cardiac cats.

Study	Location	Study type	Cats per study	Sex	Age	Breed	Average weight
Sugimoto et al. [9]	Osaka, Japan	Case–control study	5 healthy	2 males, 3 females	4–7 years	SRD	3.8 kg
Oldach et al. [6]	California, United States	Case–control study	21 (CHF) and 7 (healthy)	21 males, 7 females	6.5 years (HCM) and 3.5 years (healthy)	SRD, Himalayan, Maine Coon, Siamese	5.8 kg (CHF) and 5.5 kg (healthy)
Schober et al. [11]	Ohio, United States	Case–control study	76 CHF	58 males, 18 females	6.5 years	Various	5 kg
Kochie et al. [7]	Ohio, United States	Prospective clinical cohort study	22 (HCM) and 11 (healthy)	22 males, 11 females	7 years (HCM) and 6 years (healthy)	SRD, Sphynx, Siamese	5.73 kg (HCM) and 4.82 kg (healthy)
Fujii et al. [8]	Kanagawa, Japan	Case–control study	6 healthy	3 males, 3 females	4–7 years	SRD	3.85 kg
Ward et al. [4]	Iowa and North Carolina, United States	Retrospective cohort study	260 ICC	187 males, 73 females	10 years	SRD, Maine Coon, Siamese, Sphynx, Himalayans, Persians, Others	5.1 kg
Oldach et al. [19]	California, United States	Case–control study	13 CHF	Not reported	Not reported	Not reported	Not reported
Gordon et al. [12]	Texas, United States	Retrospective cohort study	27 ICC	16 males, 11 females	7 years	SRD, Sphynx, Maine Coon, Others	2.7 kg
Hanzlicek et al. [3]	Kansas, United States	Case–control study	18 healthy	14 males, 4 females	Young adults	Not reported	5.2 kg
MacGregor et al. [13]	Massachusetts, United States	Retrospective cohort study	170 ICC	117 males, 53 females	11 years	SRD, Maine Coon, Siamese, Persians, Others	5 kg

*Note:* CHF, congestive heart failure; SRD, mixed breed; HCM, feline hypertrophic cardiomyopathy.

A total of 636 domestic cats participated in the treatment with pimobendan, with 47 healthy animals and 589 sick animals. Only two articles used healthy cats as a control group [6, 7] to compare with sick cats, while three studies focused solely on healthy cats to evaluate the drug’s tolerability and effects on the species [3, 8, 9].

Among the diseases, feline hypertrophic cardiomyopathy (HCM) was the most commonly reported (6/10). Some authors used exclusively cats with HCM [6, 10, 11], while others also included cats with other cardiac diseases, provided they had congestive heart failure (CHF) [4, 12–14]. One study specifically included cats with dilated cardiomyopathy (DCM), arrhythmogenic right ventricular cardiomyopathy (ARVC), and congenital heart diseases, in addition to HCM [13].

Among the total animal population included in the studies (636), 440 were male and 183 were female. One study did not report the sex of the animals included [10], so it is unclear whether the remaining 13 animals were male or female.

The average age of the animals was 7.6 years for cardiopathic patients and 5 years for healthy cats; however, one study did not provide the age of the cats [10], and another described them as young adults without specifying the exact age [3]. Regarding breeds, one study did not report the breed of the included animals (*n* = 13) [10], while the other studies showed considerable variability. The most common breeds were domestic shorthair (DSH) (371/636), Maine Coon (25/636), Siamese (21/636), Ragdoll (12/636), Persian (8/636), and 11 other breeds (199/636).

The average weight of the sick animals was 4.68 kg, and the healthy ones weighed 4.7 kg. However, one article did not report the weight of the patients [10]. It is noteworthy that the weight varied greatly between the groups and animals in the studies, with the lowest being 1.3 kg and the highest 10.5 kg. Animals included in the studies, with body weights ranging from 1.3 kg to 10.5 kg.

The dosage, observed effects, and adverse effects are presented in Table 2. The dose varied between the standard per cat (commercial recommendation) and mg/kg. Two studies used the same protocol of 0.5 mg/kg (high dosage), 0.25 mg/kg (standard dosage, with the dog dose as a reference), and 0.125 mg/kg (low dosage) [8, 9]. Moreover, another study used the same standard dose mentioned above [7]. Three articles applied the dose of 1.25 mg/cat [4, 6, 10], and the authors emphasized that this would be a limitation of the study, as there was variability in the weight of the animals, and the same dose was used. Other dosages such as 0.3 mg/kg [11], 0.24 mg/kg [13], and an intermediate dose of 0.25–0.3 mg/kg [12] were also described.

**TABLE 2:** Main characteristics of the posology and observed effects of pimobendan in healthy and cardiopathic domestic cats.

Study	Groups	Dose	Route	Frequency	Duration	Cardiac effects	Adverse effects
Sugimoto et al. [9]	Healthy	0.5 mg/kg, 0.25 mg/kg, and 0.125 mg/kg	Oral	12 h	14 days	Increase in heart rate (HR) and fractional shortening (FS)	Not reported
Oldach et al. [6]	HCM and healthy	1.25 mg/cat	Oral	Single dose (90 min before evaluation)	Single dose	Improvement in left atrial ejection fraction (LAF) and diastolic and systolic function, tachycardia	Not reported
Schober et al. [11]	HCM and CHF	0.3 mg/kg	Oral	12 h	180 days	Severity of obstruction decreased	Lethargy, tremors, loss of appetite, fluctuating appetite
Kochie et al. [7]	HCM and healthy control group	0.25 mg/kg	Oral	12 h	4–7 days	Improvement in FS in left atrial and ventricular systolic function	Not reported
Fujii et al. [8]	Healthy	0.5 mg/kg, 0.25 mg/kg, and 0.125 mg/kg	Oral	12 h	15 days	Sinus tachycardia and idioventricular/idiojunctional complexes and increased cardiac output	Not reported
Ward et al. [4]	CHF	1.25 mg/cat	Oral	12 h	137 days	No change in hemodynamic parameters	Hyporexia/anorexia, lethargy, vomiting, diarrhea, ventricular arrhythmias, and hypotension
Oldach et al. [19]	HCM	1.25 mg/cat	Oral	Single dose (60 min before)	Single dose	Increased left atrial FS	Vomiting, diarrhea, agitation, salivation, and ventricular tachycardia
Gordon et al. [12]	CHF	0.25–0.30 mg/kg	Oral	12 h	Continuous	No change	Hypotension
Hanzlicek et al. [3]	Healthy	0.28–0.31 mg/kg	Oral	24 h–12 h	7 days	Not reported	Vomiting, salivation
MacGregor et al. [13]	Advanced and refractory CHF	0.24 mg/kg	Oral	12 h	Variable	Bradyarrhythmias and tachyarrhythmias	Agitation, anorexia, vomiting, and constipation

*Note:* HCM, hypertrophic cardiomyopathy; CHF, congestive heart failure. *h*, hour.

Abbreviations: EF, ejection fraction; FS, fractional shortening; HR, heart rate; LA, left atrium; LV, left ventricle; PO, oral route; SD, single dose.

The route of administration was unanimous among the studies, where all used the oral route. The frequency of administration was every 12 h, used by eight authors [3, 4, 7–9, 11–13], and two administered only a single dose of pimobendan, and what varied was the time for reevaluation of the parameters; one was after 90 min [6] and the other after 60 min [10].

The duration of the treatment varied between authors from just one application [6, 10], four to seven days [7], seven days [3], 14 days [9], 15 days [8], an average of 137 days [4], 180 days [11], continuous [12], and variable [13].

Regarding the cardiovascular findings, two studies did not report them [3, 4]. The most common finding was an increase in the shortening fraction (6/10), followed by tachycardia (4/10), improvement in systolic function (2/10), bradycardia (1/10), reduction in dynamic left ventricular outflow tract obstruction (ODVSVE) (1/10), increased cardiac output (1/10), improvement in dyspnea and anorexia (1/10), improvement in diastolic function (1/10), among others (Table 2).

Adverse effects were not described in some studies [6–9]; however, the others reported several, such as anorexia (5/10), vomiting (4/10), lethargy (2/10), hypotension (2/10), ptyalism (2/10), agitation (2/10), diarrhea (2/10), worsening dynamic left ventricular outflow tract obstruction (1/10), arrhythmia (3/10), intermittent tremors (1/10), periuria (1/10), and constipation (1/10).

Certain studies (4/10) reported pharmacological associations where the cats were already under treatment or occasionally needed to add to the therapy, such as clopidogrel (4/4), benazepril and enalapril (3/4), furosemide (2/4), spironolactone (2/4), diltiazem (1/4), atenolol (1/4), sotalol (1/4), digoxin (1/4), hydrochlorothiazide (1/4), aspirin (1/4), and heparin (1/4) [6, 11–13].

### 3.2. Risk of Bias in Individual Studies

A risk of bias was observed in one of the studies for presenting a lack of detailed methodology regarding weight, sex, cardiovascular effects, and adverse effects [10]. Also, due to the heterogeneity in the studies regarding the cardiac diseases in which pimobendan was used, separating them into distinct groups, thus, four studies used as a parameter only signs of CHF, regardless of etiology, pathophysiology, and clinical severity [11–13]. Additionally, these same authors used pimobendan only in stage *D* patients, that is, the drug was added when the patient was refractory to conventional treatment and presented severe cardiac changes.

Another bias is that three studies treated only healthy cats to observe the tolerability and effects of pimobendan [3, 8, 9]; another two articles with a control group of healthy cats and another with HCM [6, 7]; and another only with cats diagnosed with HCM [10].

## 4. Discussion

The included studies evaluated different feline populations, including healthy cats, cats with naturally occurring cardiovascular disease, and studies comparing healthy control cats with cats affected by cardiac disease. This distinction should be considered when interpreting the available evidence, as physiological responses and clinical outcomes may vary according to the underlying cardiovascular status.

This systematic literature review presents results from electronic and printed sources, with no restrictions regarding language or publication year. However, the exclusion of works published in conference proceedings and other types of gray literature can be considered a limitation, given the shift in perspective toward these sources, even though they remain considered temporary resources or unreliable, as they often demonstrate methodological flaws [14]. The majority of the articles used in this systematic review were produced in the United States of America, and fewer were from Japan, likely due to the disparity in scientific development between countries, with the United States and Japan being among the top four most developed nations and, consequently, producing more research.

Notably, during the Mendeley screening, most studies were excluded for being about other species or subjects, despite specific search terms, showing that this topic has not been widely reported or clarified [9]. It is observed that pharmacological studies of pimobendan in cats are not as widely researched as in dogs, which have a broad body of literature and publication distribution [10].

Case–control, retrospective, and prospective cohort studies involve following a group of individuals over time to examine the association between exposure and outcomes. They offer the advantage of allowing the evaluation of various exposure factors and outcomes (primary and secondary) but present some biases regarding selection, researcher memory, and information [15].

It was observed that most of the selected articles were developed with patients with various heart diseases, especially with HCM (5/10), possibly because this disease is the most prevalent in this species, characterized by focal or generalized thickening of the left ventricle [16]. Therefore, the use of pimobendan is more restricted to cases of HCM without LVOT obstruction, where there is no dynamic outflow obstruction that could potentially be exacerbated by its inotropic effects [16]. However, the previous contraindication for the use of pimobendan in cats with LVOT obstruction has been reconsidered, and its use is generally accepted in cases of mild to moderate obstruction, with greater caution recommended in severe cases [17].

Three studies included patients with any heart disease but who were treated with pimobendan; being retrospective studies, they probably aimed to generate a larger population size and provide an overview of its effects in various conditions. A minority used healthy cats, raising the issue that the current dose for felines is still based on the dosage determined for dogs due to limited information about its cardiovascular effects, especially in healthy felines [18].

There was a higher predisposition in the selected animals, across all selected studies, to male, mature adult cats with an average age of seven years and mixed breeds, also showing a lower survival rate in this sex [12]. Literature describes some breeds as predisposed to developing cardiomyopathies, especially Maine Coons, Ragdolls, Persians, Sphynxes, and Bengals, among others, but mixed‐breed cats are the most prevalent [16], a fact corroborated by the findings of this review. The weight varied greatly among individuals, showing no pattern or predisposition concerning body score, which is a problem when using a fixed dose of the drug per animal.

The usual route for pimobendan is oral, which was unanimous among the selected articles, as well as among other authors, including in the consensus on the treatment of feline cardiomyopathies [16, 19]. Another route recently reported in the literature is intravenous, which has not yet been approved for use in cats in many countries and is still under study [17]. Since 2013, an intravenous formulation has been available in the United Kingdom for dogs with CHF, but only two articles have described its use in felines since then [17, 20]. In the limited available reports, parenteral administration did not cause adverse effects or arrhythmias in five healthy DSH cats without comorbidities, with only an increase in heart rate and paroxysmal ventricular tachycardia being recorded in a single animal treated with 0.5 mg/kg every 12 h; however, a direct causal relationship could not be established [17].

The frequency of administration every 12 h is recommended by manufacturers and the literature for the treatment of cats with CHF [16, 21]. It should be noted, however, there is a contraindication for using pimobendan: to date, pimobendan is only formally approved in the manufacturer’s labeling for use in dogs, and its application in cats is based on published literature and remains off‐label. However, exceptions that used a single dose [6, 10] were experimental studies to assess the acute pharmacodynamics in cats with subclinical and clinical cardiomyopathy and not as a form of treatment or prolonged use, differing from the others. Furthermore, pimobendan has an accumulative effect on hemodynamics [3].

There was no standardization in the duration of treatment across the included studies. However, the six studies that employed the drug for short periods (single dose up to 15 days) involved healthy cats or cats with subclinical HCM or mild CHF. In contrast, studies with longer treatment durations (ranging from 137 days to continuous administration) included cats with more advanced or decompensated cardiac disease and/or those refractory to conventional therapy without pimobendan, with follow‐up duration varying according to the clinical course of the individual patients. When compared with other retrospective or case–control studies reporting longer follow‐up periods (up to 626 days) [5], it is important to note that none of the included studies were designed to evaluate survival or long‐term clinical outcomes as primary endpoints. Therefore, although differences in treatment duration and clinical context were observed across studies, these data do not allow any inference regarding long‐term efficacy or survival outcomes of pimobendan in cats.

The doses of pimobendan used varied according to the species‐specific standard of 1.25 mg/cat [16] and doses based on dogs, ranging from 0.25 to 0.5 mg/kg [21]. Regardless of the dosage, nine studies described pimobendan as tolerated, with adverse effects being uncommon and generally not clinically relevant, even with variability in weights and even in patients with LVOT obstruction, which was previously cited as contraindicated, showing improvement in the same [4, 10], corroborating the literature [5, 16, 22]. However, it was observed that felines with HCM who have LVOT obstruction may show decreased SBP and promote hypotension [12].

However, in one study, there was a discrepancy [11], where no improvement was observed in the condition of felines treated with pimobendan or not, possibly due to some individual variation in pharmacokinetic parameters or the dose being too low, knowing that dogs should receive higher doses as the condition worsens and thus see positive responses. Although 1.25 mg/cat is a frequently used clinical dose for the drug, the variation in body weight among patients makes dose adjustments in mg/kg necessary, potentially causing different effects depending on the dose administered to each animal [6].

Regarding the observed effects, they are mainly related to improvement in cardiac output and left atrial/ventricular function, without conclusive evidence regarding a reduction in thrombus formation risk. That is, with the improvement in LA function, there would be more efficient ventricular filling and limited atrial dilation, reducing the chances of thromboembolic diseases, improving prognosis and survival for these felines [6]. Another factor is that pimobendan is a phosphodiesterase inhibitor, not a thrombus‐forming agent, meaning it also has antithrombotic action, preventing platelet aggregation [19]. Additionally, it reduces the severity of the left ventricular outflow tract obstruction [11], opposing the literature that claims it is contraindicated due to worsening obstruction [16].

Arrhythmia (ventricular/junctional rhythm, ventricular/junctional escape complexes, extrasystoles, and tachycardia) was one of the main adverse clinical signs reported in studies evaluating the use of pimobendan. Although arrhythmias are a recognized clinical concern in human patients treated with this drug, pimobendan has not demonstrated clinically relevant pro‐arrhythmic effects in dogs [12]. In cats, the available studies have not consistently established a causal relationship between pimobendan administration and the occurrence of arrhythmias, and most did not perform continuous electrocardiographic (Holter) monitoring [6, 8, 10]. Pimobendan has positive inotropic effects by sensitizing intracellular calcium (Ca^2+^) myofilaments without increasing intracellular Ca^2+^ levels or myocardial oxygen consumption. Therefore, it improves contractility without causing myocardial hypoxia [17]. Tachycardia may be observed due to dysfunction in Ca^2+^ signaling regulation in HCM or other diseases in which the myocardium is compromised [17].

All studies that highlighted gastrointestinal (GI) adverse signs involved cardiopathic felines, so if the symptoms were associated with a recurrence of CHF, medication changes, diet or resolved spontaneously, they were considered not related to the drug [4]. Additionally, it is not possible to determine whether the GI effects were directly caused by the medication or other factors, such as management, sedation, environmental changes, or stressors, since all cats maintained their usual behaviors, including appetite, social interaction, urination, and defecation [3]. Another hypothesis supporting this is that the use of parenteral pimobendan did not demonstrate adverse effects like oral administration [17].

A limitation of this systematic review was the heterogeneity of the studied population, with several heart diseases treated with pimobendan, using only CHF signs without considering etiology and clinical severity. Additionally, some studies obtained using pimobendan involved only healthy cats, along with dose variability, making data comparison difficult [6].

## 5. Conclusion

Pimobendan was tolerated at doses of 0.24–0.5 mg/kg or 1.25 mg/cat, with only isolated adverse effects and no consistent clinically relevant alterations reported. The recommended dosage is oral administration every 12 h, which may be associated with infrequent and transient adverse effects; however, it is not possible to determine whether these were directly caused by the medication or by other factors, since the parenteral formulation did not demonstrate adverse effects.

## Funding

This study was supported by the Coordenação de Aperfeiçoamento de Pessoal de Nível Superior.

## Conflicts of Interest

The authors declare no conflicts of interest.

## Data Availability

Data sharing is not applicable to this article, as no datasets were generated or analyzed during the current study.
